# Ultrasonographical, clinical and histopathological features of 1264 nodules with papillary thyroid carcinoma and microcarcinoma based on tumor size

**DOI:** 10.20945/2359-3997000000286

**Published:** 2020-08-24

**Authors:** Neslihan Cuhaci Seyrek, Husniye Baser, Oya Topaloglu, Didem Ozdemir, Aydan Kilicarslan, Reyhan Ersoy, Bekir Cakir

**Affiliations:** 1 Ankara Yildirim Beyazit University Faculty of Medicine Ataturk Education and Research Hospital Ankara Turkey Ankara Yildirim Beyazit University, Faculty of Medicine, Ataturk Education and Research Hospital, Department of Endocrinology and Metabolism, Ankara, Turkey; 2 Ankara Yildirim Beyazit University Faculty of Medicine Ataturk Education and Research Hospital Ankara Turkey Ankara Yildirim Beyazit University, Faculty of Medicine, Ataturk Education and Research Hospital, Department of Pathology, Ankara, Turkey

**Keywords:** Papillary thyroid carcinoma, papillary thyroid microcarcinoma, thyroid ultrasonography, histopathology, multifocality

## Abstract

**Objective:**

We aimed to evaluate the patients diagnosed with papillary thyroid carcinoma (PTC) and papillary thyroid microcarcinoma (PTMC) in terms of clinical, ultrasonographical (US) and histopathological features and their relationships with tumor size.

**Subjects and methods:**

We retrospectively evaluated 881 patients who underwent thyroid surgery in our clinic and diagnosed with PTC histopathologically were enrolled the study. Demographic characteristics, US findings and histopathological features were evaluated.

**Results:**

In total, 1264 nodules were identified in the 881 patients. The incidentality rates were higher in the PTMC group and also in the ≤ 5 mm group. In total multifocality rate was 32.9%, and was significantly higher in PTMC group than the PTC group. PTC and > 5 mm PTMC groups compared to PTMC and ≤ 5 mm groups respectively, were more aggresive histopathological features.

**Conclusions:**

Since the incidentality rates were found significantly more common in our patients with PTMC and those with ≤ 5 mm, ultrasonographic features of the nodules should be evaluated carefully and for cases which are suspicious with US, US-guided fine needle aspiration biopsy (FNAB) should be considered in order to make the correct treatment strategy. Also our study revealed that PTC and > 5 mm PTMC groups compared to PTMC and ≤ 5 mm groups respectively, have more aggresive histopathological features.

## INTRODUCTION

Papillary thyroid carcinoma (PTC) is the most common type of thyroid cancer, with an increasing incidence in the last decade (1). PTC usually proceeds slowly and has a good prognosis if resected sufficiently (2). PTC with a diameter of ≤ 10 mm is defined as papillary thyroid microcarcinoma (PTMC) by the World Health Organization (WHO) (3). In most populations worldwide, thyroid cancer rates have been increasing, and 49% of this increase is due to PTMC (4). In recent years, the increasing prevalence of PTMC has been attributed to not only the increasing use of high-resolution ultrasonography (US) and US-guided fine-needle aspiration biopsies (FNAB) (4,5), but also an actual increase in tumor occurrence (6) and the increased accuracy of pathological thyroid examinations, particularly with the number and thinness of anatomical slices (7).

In most patients, PTMC has a slow progression (3) and many studies have reported that it is an indolent disease with an excellent prognosis and low mortality rate (8). Moreover, the higher incidence of PTMC in autopsy studies suggests that most PTMCs are benign (9). However, some PTMC cases have aggressive features such as local lymph node metastasis, extrathyroidal extension (ETE), loco-regional recurrence, and distant metastasis (10–12). Thus, the clinical significance of PTMC remains unclear (5). Because PTMC is being diagnosed at increasing rates, it is important to determine the clinical and pathological factors that contribute to its aggressive nature. In recent years, due to the improvements in US technique, it has become possible to gain more information about the tumor (2). Nonetheless, little is known about whether and how the US features of PTMC reflect biologically aggressive phenotypes (2). US features that suggest malignancy in a thyroid nodule include microcalcifications, absence of a halo sign, marked hypoechogenicity, ETE, an irregular or microlobulated margin, and a solid texture (13). However, whether PTC and PTMC exhibit the same US features is controversial (1).

In this study we aimed to evaluate clinical, ultrasonographical and histopathological features and their relationships with tumor size in PTC and PTMC. We also compared clinicopathological features in patients with PTMC ≤ 5 mm and > 5 mm sizes.

## SUBJECTS AND METHODS

### Patients

We retrospectively evaluated 881 patients diagnosed with PTC histopathologically in our clinic. Operation indication were; giant nodule, hyperthyroidism (toxic multinodular goitre, toxic adenoma or Graves’ disease), and FNAB results (malignancy/suspicious of malignancy/atypia of undetermined significance or follicular lesion of undetermined significance with suspicious US findings/non-diagnostic). Patients with a previous history of thyroid or parathyroid surgery, previous neck surgery, percutaneous interventions, radiotherapy of the head and neck, and other types of thyroid malignancy including poorly differentiated thyroid carcinoma, medullary thyroid carcinoma, and thyroid lymphoma were excluded.

Demographical data, preoperative thyroid functions, thyroid autoantibodies, US findings and histopathological features were evaluated. Local ethical committee approval was obtained in accordance with the ethical standards of the Helsinki Declaration.

### Laboratory findings

Levels of thyroid-stimulating hormone (TSH), free triiodothyronine (fT3), free thyroxine (fT4), thyroid autoantibodies (thyroid peroxidase antibody [anti-TPO] and thyroglobulin antibody [anti-Tg]), and thyroglobulin were measured in all patients using chemiluminescence methods (Immulite 2000; Diagnostic Products Corp., Los Angeles, CA and UniCel DxI 800; Beckman Coulter, Brea, CA). The normal ranges for TSH, fT3, fT4, anti-Tg, and anti-TPO were 0.4–4 μIU/mL, 1.57–4.71 pg/mL, 0.61–1.12 ng/dL, < 30 U/mL, and < 10 U/mL, respectively.

### Conventional US and fine-needle aspiration biopsy

All patients underwent US preoperatively. Thyroid US was performed with an US device (General Electric Logiq pro 200, Model number: 2270968, GE Healthcare Korea, Seongnam-SI, Gyeon GGI-DO, Korea) and a 5.5-7.5 MHz probe between 2007 and 2010, with Esaote color Doppler US (Model 796FDII; MAG Technology Co. Ltd., Yung-Ho City, Taipei, Taiwan) and a standard US with a superficial probe (Model LA523 13-4, 5.5-12.5 MHz) between 2010-2012 and with Hitachi EUB 7500 elastosonography (Hitachi Medical Corporation 4-14-1, Soto-Kanda, Chiyoda-ku, Tokyo, Japan) and 3 different superficial probes that were compatible with this machine between after the year 2012. None of the researchers of this study was a member or consultant of Hitachi Medical, General Electric or MAG Technology. The diameter (mm), texture, echogenicity, border regularity, microcalcification, macrocalcification and halo sign of the nodules were evaluated. The echogenicity of the nodule was compared to that of the surrounding parenchyma and was classified as hypoechoic, isoechoic or isohypoechoic. The nature of the nodule was classified as solid (solid component >50%), mixed (containing a cystic part), or pure cystic (almost cystic or very little solid part). Nodules of ≥ 1 cm or < 1 cm with suspicious US features, such as an irregular border, hypoechoic texture, solid component, presence of microcalcification, and anteroposterior/transverse diameter > 1 were defined as suspicious US features and underwent FNAB (14). FNAB was performed under US guidance using a General Logic Pro 200 system (model 2270968; GE Healthcare, Korea and Seongnam SI; Gyeonggi-do, Korea) and a 5.5–7.5 MHz superficial probe. No anesthetic was used before the procedure. For all patients, US-guided FNAB was performed by an experienced endocrinologist using a 23-gauge needle and 20 mL syringe. Written consent was obtained from all patients prior to FNAB. All the FNAB samples were performed in the euthyroid state.

### Cytological and histopathological examinations

Materials obtained by US-guided FNAB were air-dried, stained with May-Grünwald-Giemsa, and re-evaluated according to the Bethesda system classification (15). The cytology results were as follows: 1) non-diagnostic, 2) benign, 3) atypia/follicular lesion of undetermined significance (AUS/FLUS), 4) follicular neoplasm/suspicious for follicular neoplasm, 5) suspicious for malignancy and 6) malignant (16,17).

Histopathological evaluation was made according to the 2004 WHO criteria (18). The diagnosis of PTC was based on nuclear features, such as enlarged and elongated nuclei with an irregular contour, chromatin clearing with peripheral margination of chromatin, nuclear grooves, and intranuclear cytoplasmic pseudoinclusions. According to the WHO criteria, the PTC diagnosis was confirmed if the tumor diameter was > 10 mm, while PTMC was confirmed with a tumor diameter of ≤ 10 mm (3).

### Statistical analysis

Data analysis was performed by using SPSS for Windows, version 17.0 (SPSS Inc., Chicago, IL, United States). Whether the continuous variables were distributed normally or not was determined by Kolmogorov Smirnov test. While, the continuous data were shown as mean ± standard deviation (SD) or median (min-max), otherwise, number of cases and percentages were used for categorical variables. Differences between the means of different groups were evaluated by Student's *t*-tests, and differences in median values were evaluated by Mann–Whitney *U*-tests. Categorical data were analyzed by Pearson's Chi-square, Fisher's exact or Likelihood Ratio test, where appropriate. A p value less than 0.05 was considered statistically significant.

## RESULTS

There were 881 patients of whom 698 (79.2%) were female (the mean age was 48.57 ± 12.52), and 183 (20.8%) were male (the mean age was 50.37 ± 13.82). The mean TSH level was 1.3 mIU/mL, anti-TPO positivity rate was 20.5%, and anti-Tg positivity was 20.3%. Age, sex, thyroid function tests and thyroid antibody positivity rates of PTC and PTMC patients’ were shown in Table 1. The mean age of the patients with PTC were significantly lower than the PTMC group (p < 0.001). TSH levels of the patients with PTC were significantly higher than the patients with PTMC (p = 0.001). There were no significant differences between the groups regarding with thyroid antibody positivity (Table 1).

**Table 1:** Demographical features and laboratory findings in patients with papillary thyroid carcinoma and microcarcinoma

Variables		PTC (n = 388)	PTMC (n = 493)	p-value
Age (years)		46.68 ± 13.78	50.77 ± 11.71	**< 0.001**
Sex				0.150
	Female	316 (81.4%)	382 (77.5%)	
	Male	72 (18.6%)	111 (22.5%)	
TSH		1.94 ± 1.72	1.53 ± 1.67	**0.001**
fT3		3.29 ± 0.62	3.35 ± 1.66	0.544
fT4		1.18 ± 0.23	1.16 ± 0.39	0.513
**Thyroid antibody positivity**		**n = 306**	**n = 408**	
Anti-TPO positivity		82 (26.8%)	94 (23.0%)	0.249
Anti-Tg positivity		74 (24.18%)	102 (25%)	0.821

PTC: papillary thyroid carcinoma; PTMC: papillary thyroid microcarcinoma; TSH: thyroid-stimulating hormone; fT3: free triiodothyronine; fT4: free thyroxine; Anti-TPO: thyroid peroxidase antibody; Anti-Tg: thyroglobulin antibody.

### Ultrasonographical features of the nodules with papillary thyroid carcinoma and papillary thyroid microcarcinoma

In total, 1264 tumor foci of 881 patients were evaluated. There were 360 PTC and 904 PTMC.

After exclusion of incidental tumors, preoperative US features were available in 314 tumors in the PTC group and 221 tumors in the PTMC group. Comparison of US features of the nodules were shown in the Table 2. Irregular border were significantly more common in the PTMC group, but halo sign, microcalcifications, and macrocalcifications were significantly more common in the PTC group. In addition, isoechoic appearance was more frequent in the PTC and hypoechoic appearance was more frequent in the PTMC group (p = 0.011).

**Table 2:** Ultrasonography features of nodules with the histopathological diagnosis of papillary thyroid carcinoma and microcarcinoma

Variables		PTMC (n = 221)	PTC (n = 314)	p-value
Irregular border		152 (68.8%)	181 (57.6%)	**0.009**
Halo sign		41 (18.6%)	102 (32.5%)	**< 0.001**
Microcalcifications		90 (40.7%)	156 (49.7%)	**0.044**
Macrocalcifications		55 (24.9%)	118 (37.6%)	**0.002**
Echogenicity				**0.011**
	Isoechoic	67 (30.3%)	128 (40.8%)	
	Hypoechoic	63 (28.5%)	60 (19.1%)	
	Isohypoechoic	91 (41.2%)	126 (40.1%)	
Nodule texture				0.726
	Solid	216 (97.7%)	304 (96.8%)	
	Mixed	2 (0.9%)	4 (1.3%)	
	Cystic	3 (1.4%)	6 (1.9%)	

PTC: papillary thyroid carcinoma; PTMC: papillary thyroid microcarcinoma.

PTMC group was further classified according the tumor size; ≤ 5 mm, and > 5 mm. Of these 221 nodules 53 (24%) were in the ≤ 5 mm, 168 (76%) were in the > 5 mm group. The US features of the two groups were shown in Table 3. Irregular border were significantly more common in patients > 5 mm group than those in the ≤ 5 mm group. The halo sign was significantly more common in the ≤ 5 mm group. Micro and macrocalcifications, echogenicity and nodule texture were not significantly different between the groups (Table 3).

**Table 3:** Ultrasonography features of nodules with the histopathological diagnosis of papillary thyroid microcarcinoma ≤ 5 mm and > 5 mm

Variables		PTMC ≤ 5 mm (n = 53)	PTMC > 5 mm (n = 168)	p-value
Irregular border		26 (49.1%)	126 (75%)	**< 0.001**
Halo sign		16 (30.2%)	25 (14.9%)	**0.015**
Microcalcifications		16 (30.2%)	74 (44.0%)	0.059
Macrocalcifications		10 (18.9%)	45 (26.8%)	0.220
Echogenicity				0.333
	Isoechoic	20 (37.7%)	47 (28%)	
	Hypoechoic	15 (28.3%)	48 (28.6%)	
	Isohypoechoic	18 (34%)	73 (43.4%)	
Nodule texture				0.221
	Solid	51 (96.2%)	165 (98.2%)	
	Mixed	1 (1.9%)	2 (1.2%)	
	Cystic	1 (1.9%)	1 (0.6%)	

PTMC: papillary thyroid microcarcinoma.

### Tumor characteristics in papillary thyroid carcinoma and papillary thyroid microcarcinoma

In total, multifocality rate was 32.9%, and it was significantly higher in PTMC group than the PTC group (28.95% vs. 6.95%, respectively, p < 0.001).

Histopathological features of 904 nodules in the PTMC group and 360 nodules in the PTC group were analyzed. The rate of incidentally was 4.3% in the PTC group, whereas 73.7% was in the PTMC group (p < 0.001). Lymphatic, vascular and capsular invasion, ETE, lymph node metastasis were significantly higher in the PTC group than the PTMC group (Table 4).

**Table 4:** Histopathological features of papillary thyroid carcinoma and microcarcinoma

Variables	PTMC (n = 904)	PTC (n = 360)	p-value
Tumor diameter (mm)	4 (0,1-10)	16 (11-80)	−
Incidentality	620 (73.7%)	14 (4.3%)	**< 0.001**
Lymphatic invasion	5 (0.6%)	10 (2.8%)	**0.002**
Vascular invasion	6 (0.7%)	22 (6.1%)	**< 0.001**
Capsular invasion	101 (11.2%)	141 (39.3%)	**< 0.001**
Extrathyroidal extension	55 (6.1%)	76 (21.2%)	**< 0.001**
Lymph node metastasis	26 (5%)	46 (13.9%)	**< 0.001**
Multifocality	141 (28.95%)	18 (6.95%)	**< 0.001**

PTMC: papillary thyroid microcarcinoma; PTC: papilary thyroid carcinoma.

When subgroup analysis was made in PTMC, 591 carcinomas were in the ≤ 5 mm group and 313 were in the > 5 mm group. Multifocality rate was significantly higher in the > 5 mm PTMC group than the ≤ 5 mm group (38.9% vs 25.6%, p = 0.001). Incidentally was significantly higher in the ≤ 5 mm compared to > 5 mm group (90.5% vs 41.1%, p < 0.001). In PTMC > 5 mm, capsular invasion, ETE, and lymph node metastasis were significantly more common than ≤ 5 mm PTMC. Lymphatic and vascular invasion were similar in two groups (Table 5).

**Table 5:** Histopathological features of papillary thyroid microcarcinoma > 5 mm and ≤ 5 mm

Variables	PTMC ≤ 5 mm (n = 591)	PTMC > 5 mm (n = 313)	p-value
Tumor size (mm)	3 (0.1-5)	8 (5.5-10)	−
Incidentality	503 (90.5%)	117 (41.1%)	**< 0.001**
Lymphatic invasion	3 (0.5%)	2 (0.6%)	1.000
Vascular invasion	2 (0.3%)	4 (1.3%)	0.190
Capsular invasion	35 (6%)	66 (21.2%)	**< 0.001**
Extrathyroidal extension	22 (3.7%)	33 (10.5%)	**< 0.001**
Lymph node metastasis	7 (2.3%)	19 (8.6%)	**< 0.001**
Multifocality	81 (25.6%)	88 (38.9%)	**0.001**

PTMC: papillary thyroid microcarcinoma.

## DISCUSSION

PTC is the most common type of thyroid cancer and have a good prognosis with a low degree of malignancy (1). PTMC has an excellent prognosis with a 0–1% mortality rate (19). Some studies have reported that advanced age is a poor prognostic factor for well-differentiated thyroid carcinoma (20), in contrast another study found no effect of age on the aggressiveness or metastasis of PTMC (21). In our study age was significantly lower in patients with PTC than patients with PTMC. This may be related with the increased use of neck US in elderly patients which leads to diagnose incidental PTC and/or PTMC with a higher incidence. Also our study revealed that TSH levels of the patients with PTC were significantly higher than the patients with PTMC. It is not surprising since it is known that chronic stimulation of elevated TSH levels may lead to thyroid gland hyperplasia and finally can result with thyroid neoplasia.

There is not yet a biological marker for the prediction of prognosis and there is lack of randomized studies concerning predictive factors for cancer aggressiveness (5). Recent studies have demonstrated that US features may be useful for the prediction of the biological behavior of PTCs (3). Although many studies have sought to assess the risk of thyroid cancer attributable to certain US features and to formulate a combination of US features to increase the predictive value for malignancy, the diagnostic accuracy of US remains limited (22). Nam and cols. demonstrated that PTCs with suspicious US features (defined according to at least one of these: taller-than-wide shape, marked hypoechogenicity, microcalcifications, and infiltrative borders) had more aggressive biological behavior than PTCs lacking the criteria above (23). It has been reported that PTMC nodules are primarily hypoechoic, which is associated with a low degree of differentiation in cancer cells, fewer interstitial components, and thus good sound transmission through the tumor (1). With nodule growth, blood vessels and fibrous tissue undergo hyperplasia, which leads to variation in the echogenicity (1). When the tumor grows faster, liquefaction necrosis and cystic changes may occur (24). In the study by Zhang and cols. cystic changes were observed in PTC with more than half of the cases accompanied by microcalcifications in the solid component (1). But some studies have showed that echogenicity does not depend on nodule size (3). In our study we found that; isoechoic appearance was 40.8%, hypoechoic appearance was 19.1% and isohypoechoic appearance was 40.1% in the PTC group, whereas, the rates were 30.3%, 28.5% and 41.2% respectively in the PTMC group. Comparison of the both groups revealed that hypoechoic appearance was more frequent in PTMC than PTC group. Unlike from the study by Zhang and cols. (1) we did not found any differences between cystic component. Also we found no significant differences between PTMC ≤ 5 mm and > 5 mm with regard to echogenicity and cystic component.

An ill-defined and irregular margin in a thyroid tumor suggests malignant infiltration of the adjacent thyroid parenchyma (25). A recent study reported that irregular margins are the most accurate criterion for predicting PTCs in both small and large lesions, but particularly in large ones (26). Ito and cols. reported that 21.5% of PTMC had ill-defined edges and these cases showed a worse disease-free survival than those with well-defined edges (2). The authors suggested that ill-defined edges reflected the aggressive character of PTMC (2). Because tumor size is a prognostic factor of papillary carcinomas (27), it seems reasonable that the incidence of ill-defined edges in PTMC would be lower than that reported in larger tumors. However, in our study, irregular margins were significantly more common in patients with PTMC than patients with PTC, but also more common in the > 5 mm PTMC group compared with ≤ 5 mm PTMC group. Halo sign is caused by compression of normal thyroid tissue or thyroid capsule. Although halo sign is more frequent in benign thyroid nodules in the litherature it has been reported that halo sign was present 26% of the noules with PTC (28), and 75% of follicular variant of PTC (29). In our study, halo sign was seen in the PTC group 32.5% and 18.6% in the PTMC group. In the most of the nodules with PTC and PTMC no halo sign was seen (67.5% vs. 81.4%, respectively).

Microcalcification in neck US is one of the most specific indicators in the sonographic diagnosis of PTC (30). They have been shown in thyroid lesions to have been a high predictive value (42-94%), but a low sensitivity (26–59%) in the diagnosis of malignancy (7,30). In a study that compared the sensitivity and specificity of microcalcification in large and small nodules with a cut-off size of 1 cm, microcalcifications were more diagnostic for thyroid cancer in nodules larger than 1 cm (51.4% Sn, 91.6% Sp) than in nodules smaller than 1 cm (36.6% Sn, 87.9% Sp) (31). These findings suggest that the frequency of microcalcification was lower in PTMCs, and that in nodules of ≤ 1 cm, microcalcification was not a major predictor of malignancy (31). Other calcification types, including macrocalcifications, eggshell, and rim calcifications, have been thought to be more frequent in benign lesions than malignant lesions (32). However, calcification patterns other than microcalfications have also been observed in malignant lesions (32). Our micro and macrocalcification rates were significantly higher in the PTC group compared with PTMC (49.7% vs. 40.7% and 37.6% vs. 24.9%, respectively), but not significantly different between > 5 mm and ≤ 5 mm PTMC groups.

Multifocality has been found in 30–40% of PTMC cases (12,33). In previous studies concerning PTMCs, multiplicity was found in 15–33% of cases and bilaterality was found in 16–19% of cases (34,35). Although there are conflicting results as to whether multifocality in PTMC is associated with worse prognosis, according to a recent meta-analysis, the risk of lymph node metastasis in multifocal tumors is almost double compared to that of unifocal tumors (36). We analyzed multifocality in each group and found that multifocality was significantly higher in PTMC group than PTC group (28.95% and 6.95%, respectively) and also significantly higher in the > 5 mm PTMC group than ≤ 5 mm group.

The rate of ETE in patients with PTMC varies from 2% to 53% (19,37). Kim and cols. found that 34% of patients with PTMC had ETE (4). Although the rate was significantly higher in the > 5 mm PTMC group, ETE was present in 22.2% of patients with < 5 mm PTMC. In our study we found significantly higher ETE in PTC compared with PTMC (21.2% and 6.1% respectively). Additionally, it was significantly higher in the > 5 mm PTMC group than those with ≤ 5 mm PTMC (10.5% and 3.7% respectively). Also we found that lymphatic, vascular, capsular invasion, and lymph node metastasis was significantly higher in the patients with PTC than PTMC as expected.

There are conflicting data about the aggressiveness of PTC and PTMC also PTMC ≤ 5 mm and > 5 mm. Arora and cols. (38) revealed that the rates of aggressive features for PTMCs with lymph node metastasis and extrathyroidal extension was similar to the rates of PTCs. Therefore, they suggested that PTMCs should have been managed like PTCs. Park and cols found that the prevelance of ETE and initial node metastasis in patients with PTMC was surprisingly high and almost as high as that for patients with PTC (39). And they suggest that PTMC is not an occult cancer and it can act like larger PTC. In our study lymphatic, vascular, capsular invasion, ETE and lymph node metastasis were significantly higher in PTC group.

In one previous study, lymph node metastasis, ETE, and multifocality were more frequent in the PTMC > 5 mm than in the ≤ 5 mm PTMC group (5), whereas in another study, lymph node metastasis was also more common in the > 5 mm group but the rate of ETE did not differ (40). In another study including 2798 patients with PTCs, more frequent capsule invasion, bilaterality, and nodal metastasis was observed in PTMCs > 5 mm compared to PTMCs ≤ 5 mm (41). These findings suggest that there is no need for further evaluation of thyroid nodules < 5 mm in the clinical setting, despite the rare case of local tissue invasion in this category (8). However, in contrary to these findings, no difference in the tumor aggressiveness between patients with PTMCs ≤ 5 mm and > 5 mm were reported in some other studies (38). In another study, tumors ≥ 8 mm but ≤ 1 cm were more aggressive, but size was not an independent risk factor for aggressiveness (35). In our study, we found that capsular invasion, ETE and lymph node metastasis, were significantly more common in patients with PTMCs > 5 mm than those with ≤ 5 mm. However, lymphatic and vascular invasion were similar in both groups. The prevalence of these histopathological risk factors in patients with PTMC has increased with an increasing trend towards total thyroidectomy, as adopted in many centers, including ours.

In our study incidentally rate was significantly higher in PTMC (73.7%) than PTC cases (4.3%). Additionally, 90.5% of ≤ 5 mm PTMC cases, while 41.1% of > 5 mm PTMC cases were incidental. These rates may be attributable to factors such as widespread use of cervical US, US-guided FNAB of non-palpable thyroid nodules, and more extensive histological sampling of resected thyroids, all of which can lead to more accurate histopathological searches for smaller PTMCs (7,8). In addition, the use of total thyroidectomy for the treatment of benign thyroid diseases increases the diagnosis of PTMC in histopathological analyses (42,43). All of these factors inevitably revealed small PTMCs.

Although our institution is a highly specialized and extensive center for treating thyroid diseases in our region, the first limitation of this study is that it was a retrospective including patients from a single institution. A second limitation is that we evaluated only clinicopathological features, without long-term follow-up results, including disease recurrence and disease-free survival, for all groups. In addition, because routine central lymph node dissection was rarely performed in our center in the past years, it is not possible to exhibit any correlation between tumor size and lymph node metastasis certainly. Another limitation of our study is the lack of the US features of the incidental papillary thyroid cancers. Also, since the nodule vascularization had not been evaluated in a considerable number of patients we did not include it in the analysis. Although US is a noninvasive diagnostic method, a major limitation is that it is operator dependent and interobserver variability may be observed (44,45). Finally, the retrospective assessment of US features from reports as in our study may be a limitation for the accuracy of ultrasonographic evaluation.

In conclusion, our study revealed that PTC and > 5 mm PTMC groups compared to PTMC and ≤ 5 mm groups respectively, have more aggressive histopathological features. Although US has an important role in the detection and differential diagnosis of PTC, preoperative US features of PTC and PTMC are different. Incidentally rates were significantly more common in our patients with PTMC and those with ≤ 5 mm. Thus, US features of small nodules should be evaluated carefully and for cases with suspicious US features, US-guided FNAB should be considered in order to decide the correct treatment strategy.
